# Emergency Department Access to Buprenorphine for Opioid Use Disorder

**DOI:** 10.1001/jamanetworkopen.2023.53771

**Published:** 2024-01-29

**Authors:** Andrew A. Herring, Allison D. Rosen, Elizabeth A. Samuels, Chunqing Lin, Melissa Speener, John Kaleekal, Steven J. Shoptaw, Aimee K. Moulin, Arianna Campbell, Erik Anderson, Mariah M. Kalmin

**Affiliations:** 1 Bridge, Public Health Institute, Oakland, California; 2 Department of Emergency Medicine, Highland General Hospital–Alameda Health System, Oakland, California; 3 Department of Emergency Medicine, University of California, San Francisco; 4 Department of Family Medicine, David Geffen School of Medicine, University of California, Los Angeles; 5 Department of Emergency Medicine, David Geffen School of Medicine, University of California, Los Angeles; 6 Department of Psychiatry and Biobehavioral Sciences, David Geffen School of Medicine, University of California, Los Angeles; 7 Department of Emergency Medicine, University of California, Davis, Sacramento, California; 8 Department of Behavioral and Policy Sciences, RAND Corporation, Santa Monica, California

## Abstract

**Importance:**

Although substantial evidence supports buprenorphine for treatment of opioid use disorder (OUD) in controlled trials, prospective study of patient outcomes in clinical implementation of emergency department (ED) buprenorphine treatment is lacking.

**Objective:**

To examine the association between buprenorphine treatment in the ED and follow-up engagement in OUD treatment 1 month later.

**Design, Setting, and Participants:**

This multisite cohort study was conducted in 7 California EDs participating in a statewide implementation project to improve access to buprenorphine treatment. The study population included ED patients aged at least 18 years identified with OUD between April 1, 2021, and June 30, 2022. Data analysis was performed in October 2023.

**Exposure:**

All participants were offered buprenorphine treatment for OUD (either in ED administration, prescription, or both), the uptake of which was examined as the exposure of interest.

**Main Outcomes and Measures:**

The primary outcome was engagement in OUD treatment 30 days after the ED visit, determined by patient report or clinical documentation. The association of ED buprenorphine treatment with subsequent OUD treatment engagement was estimated using hierarchical generalized linear models.

**Results:**

This analysis included 464 ED patients with OUD. Their median age was 36.0 (IQR, 29.0-38.7) years, and most were men (343 [73.9%]). With regard to race and ethnicity, 64 patients (13.8%) self-identified as non-Hispanic Black, 183 (39.4%) as Hispanic, and 185 as non-Hispanic White (39.9%). Most patients (396 [85.3%]) had Medicaid insurance, and more than half (262 [57.8%]) had unstable housing. Self-reported fentanyl use (242 [52.2%]) and a comorbid mental health condition (328 [71.5%]) were common. Interest in buprenorphine treatment was high: 398 patients (85.8%) received buprenorphine treatment; 269 (58.0%) were administered buprenorphine in the ED and 339 (73.1%) were prescribed buprenorphine. With regard to OUD treatment engagement at 30 days after the ED visit, 198 participants (49.7%) who received ED buprenorphine treatment remained engaged compared with 15 participants (22.7%) who did not receive ED buprenorphine treatment. An association of ED buprenorphine treatment with subsequent OUD treatment engagement at 30 days was observed (adjusted risk ratio, 1.97 [95% CI, 1.27-3.07]).

**Conclusions and Relevance:**

The findings of this cohort study suggest that among patients with OUD presenting to EDs implementing low-threshold access to medications for OUD, buprenorphine treatment was associated with a substantially higher likelihood of follow-up treatment engagement 1 month later. Future research should investigate techniques to optimize both the uptake and effectiveness of buprenorphine initiation in low-threshold settings such as the ED.

## Key Points

**Question:**

What is the uptake of buprenorphine among emergency department (ED) patients with opioid use disorder (OUD), and how frequently did they engage in treatment after discharge?

**Findings:**

In this cohort study of 464 patients with OUD, 86% received buprenorphine treatment in the ED and 50% remained engaged in OUD treatment 1 month later. Buprenorphine treatment was associated with a 2 times higher likelihood of 30-day engagement in OUD treatment.

**Meaning:**

These findings suggest that buprenorphine treatment for patients with OUD presenting to the ED is likely an effective strategy to support engagement in follow-up care.

## Teaser

This cohort study examines the association between buprenorphine treatment administered to patients with opioid use disorder (OUD) in the emergency department and follow-up engagement in treatment for OUD 1 month later.

## Introduction

In 2021, opioid use disorder (OUD) contributed to more than 80 000 overdose deaths in the US, up 24% from 2020.^1^ More than 5% of overdose patients seen at an emergency department (ED) die within the year, many in the first 2 days after discharge.^2,3^ The scale and persistence of the OUD epidemic is increasing the urgency to integrate screening and treatment for OUD into mainstream emergency medicine practice.^4^ Emergency departments are uniquely positioned to contribute toward improved care at all stages of the OUD treatment cascade,^5^ particularly by improving access to medication for OUD, such as buprenorphine, through direct administration or prescription. Buprenorphine treatment of OUD improves quality of life and reduces both overdose risk and mortality. Additionally, buprenorphine decreases high-risk behaviors linked to transmission of HIV and hepatitis C.^6^ Despite the many well-established benefits of buprenorphine treatment for OUD, adoption in most EDs remains limited.

There is an urgent need to roll out large-scale interventions to reduce opioid-related overdose deaths. Although results from controlled trials of ED-initiated buprenorphine are promising, studies of patient outcomes from clinical implementation of low-threshold access to buprenorphine treatment in EDs are lacking.^7,8^ A substantial effect of ED buprenorphine access on subsequent OUD treatment engagement could potentially help set benchmarks for buprenorphine delivery to ED patients with OUD, enhance clinical decision-making, and guide policy efforts aimed at optimizing the ED’s role in addressing the opioid crisis. Therefore, this study examined the association of ED buprenorphine treatment (including buprenorphine administration, prescription, or both) with subsequent OUD treatment engagement at 30 days after the ED visit.

## Methods

### Study Design and Setting

**Figure 1.**
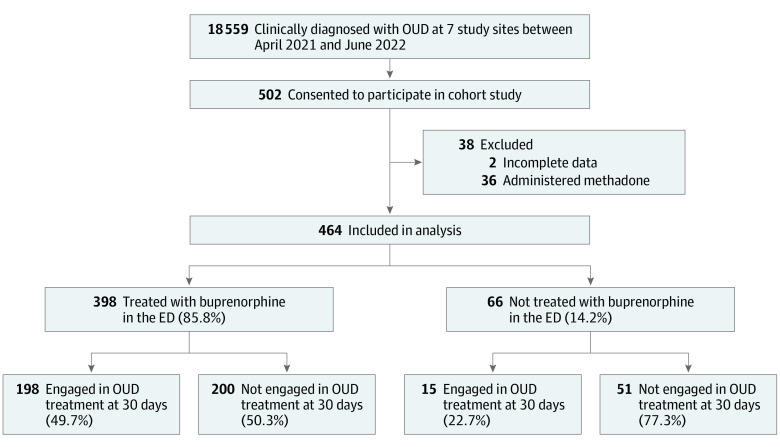
Enrollment and Flow Diagram for the CA Bridge Patient Outcomes Cohort Study Includes all emergency department (ED) visits with a discharge diagnosis of opioid use disorder (OUD) during the study period between April 1, 2021, and June 30, 2022, at all sites participating in the study. Visits that occurred during evenings and other times when research staff were not available are included.

The CA Bridge Patient Outcomes Study was conducted from April 1, 2021, to June 30, 2022. This multisite cohort study of ED patients with OUD was performed at 7 hospitals in California participating in the CA Bridge, an implementation program of the Public Health Institute (PHI) that promotes low-threshold buprenorphine treatment, care navigation, and harm reduction as described previously.^9^ This study was funded to enroll approximately 500 of the 18 559 patients identified with OUD by CA Bridge at the study sites during the time frame of interest (E.A. Samuels, MD, MPH, et al., unpublished data, 2023; Figure 1). The institutional review boards of PHI and the California Health and Human Services Agency approved this study. Patients provided written informed consent. The study followed the Strengthening the Reporting of Observational Studies in Epidemiology (STROBE) reporting guideline.

### Eligibility and Enrollment

Per CA Bridge guidelines, all sites posted publicly visible signs that advertised the availability of treatment for OUD in the triage and waiting room areas. All front-end staff were recommended to be trained in motivational interviewing and harm-reduction principles as strategies to encourage patients to openly communicate concerns around their opioid use or interest in treatment. Suspected OUD was confirmed by the attending ED physician, and all patients (regardless of participation in the cohort study) were offered both buprenorphine treatment and a consultation with a patient navigator.^9^ Participant recruitment for the cohort study was conducted during regular business hours (approximately 9:00 am to 6:00 pm, Monday through Friday). Patients were eligible for inclusion in the cohort study if they were aged at least 18 years, had OUD as confirmed by the attending ED physician, were offered CA Bridge services, and agreed to provide informed consent to participate. Potentially eligible patients were then referred to research staff who confirmed the inclusion criteria and obtained written informed consent. Patients were excluded from participation in the cohort study if they could not speak English or Spanish, could not provide consent, or were incarcerated.

### Data Collection

Participation in the cohort study involved abstraction of relevant data from the electronic health record (EHR), administered surveys that included structured assessments, and subsequent linkage to data reported to the California Department of Health Care Services (linkage data unavailable for this analysis). Standardized data forms were used to abstract relevant data (sociodemographics, substance use history, OUD treatment history, and clinical encounter information) from the EHR at baseline (ED visit) and at 7 and 30 days after the ED visit. Participants were administered surveys on clinically relevant information (including self-identified race and ethnicity) at baseline during the ED visit and at 30 days through a telephone call with research staff. For the 30-day surveys, research assistants attempted to contact all study participants. These attempts included multiple telephone calls starting at 25 days and continuing to 37 days after the ED visit for those participants who initially did not respond.

The eAppendix in Supplement 1 describes the data source for key variables. Race and ethnicity was included to assess for potential disparities in treatment uptake and patient outcomes. These data were categorized as Hispanic, non-Hispanic Black (hereinafter Black), non-Hispanic White (hereinafter White), or other race or ethnicity (American Indian or Alaska Native, Asian, Native Hawaiian or Other Pacific Islander, or multiple races or ethnicities). Unstable housing was defined as not having one’s own house, apartment, or shared housing, as measured by self-report or documentation in the participant’s EHR. A blinded researcher abstracted data for 10% of the medical records (n = 53), and there was 97.1% agreement with almost perfect interrater reliability (κ = 0.95).

### Measures

Consistent with prior studies conducted in EDs concerning medication treatment for individuals with OUD, the primary exposure, ED buprenorphine treatment, was defined as either buprenorphine administration or prescription during the ED visit.^8^ The primary outcome was OUD treatment engagement at 30 days (allowable range, 25-37 days) after the ED visit as determined by a composite measure of any or all of the following: participant self-report, EHR documentation of an active (non-ED clinician) prescription, or documentation of attendance at an OUD treatment visit. Participants were asked about their use of buprenorphine and engagement in treatment during the 30-day survey telephone call with research staff. Then the EHR was abstracted using standardized forms for indication of treatment engagement including an active buprenorphine prescription. The prescription drug monitoring program was not directly queried. However, at all sites, the ED clinical team (patient navigator and ED physician) maintained a standard practice of querying the prescription drug monitoring program to assess engagement in care after discharge for ED patients treated with buprenorphine. Any buprenorphine prescription that was filled was then documented in the EHR. Thus, the EHR data abstraction would include such documentation. Any participants lost to follow-up, defined as both no response to the 30-day follow-up telephone call and no EHR clinical documentation following the ED visit, were classified as not engaged in OUD treatment at 30 days.

### Statistical Analysis

Participant characteristics were described using frequencies for categorical variables and medians (IQRs) for continuous variables, compared by ED buprenorphine treatment status. A Poisson regression was used to assess the association of ED buprenorphine treatment with OUD treatment engagement at 30 days. Modified Poisson regression yields a direct estimate of the risk ratio by fitting a Poisson regression model with a binary outcome; all individuals were assigned a value of 1 for their time at risk, because this was a cohort study in which all participants were followed for the same amount of time.^10,11^ Robust sandwich estimators were used to produce valid variance estimates, as Poisson regression with a binary outcome has been shown to yield overestimated variances.^10^ To account for hospital-level effects, the site was included as a group-level random effect. Modified Poisson regression was chosen over log-binomial regression due to convergence issues that may arise with log-binomial regression, particularly when estimating an adjusted risk ratio.^10^

The bivariate association between the exposure and the outcome of interest was assessed first, and then an adjusted model was used to account for relevant confounders determined a priori. These confounders included age, sex, race and ethnicity, housing status, acceptance or availability of a patient navigator during the ED visit, and prior buprenorphine exposure (either EHR documented or self-reported). As a logical next step to disentangle the components of the bundled ED buprenorphine treatment intervention, the frequencies of patients were estimated for those who were both administered and prescribed buprenorphine, administered buprenorphine only, prescribed buprenorphine only, and neither administered nor prescribed buprenorphine. A χ^2^ test was used to compare each treatment component by 30-day treatment engagement. *P* < .05 (2-tailed) was considered statistically significant. Statistical analysis was conducted with R, version 4.2.1 (R Project for Statistical Computing). Data analysis was performed in October 2023.

## Results

After 2 individuals who had incomplete data and 36 who were administered methadone were excluded, 464 participants were included in this analysis (Figure 1). The median age of participants was 36.0 (IQR, 29.0-38.7) years, and 343 (73.9%) were men and 121 (26.1%) were women. With regard to race and ethnicity, 64 participants (13.8%) were Black, 183 (39.4%) were Hispanic, and 185 were White (39.9%). Most participants were Medicaid enrollees (396 [85.3%]), reported unstable housing (262 [57.8%]), and had a comorbid mental health condition (328 [71.5%]). Self-reported use of fentanyl (242 [52.2%]) and methamphetamine (232 [50.0%]) was common (Table 1). Interest in buprenorphine treatment was high: 398 patients (85.8%) received buprenorphine treatment; 269 (58.0%) were administered buprenorphine in the ED and 339 (73.1%) were prescribed buprenorphine.

**Table 1.** Baseline Characteristics of CA Bridge Patient Outcomes Study Participants^a^

Characteristic	Total cohort (N = 464)^b^	Received ED buprenorphine treatment^c^	
		Yes (n = 398)	No (n = 66)
No. of participants per site, median (IQR)	75.0 (44.0-91.0)	63.0 (37.0-81.5)	12.0 (2.0-13.0)
Age, median (IQR), y	36.0 (29.0-38.7)	35.0 (29.0-38.2)	41.0 (32.0-41.6)
Sex			
Male	343 (73.9)	295 (74.1)	48 (72.7)
Female	121 (26.1)	103 (25.9)	18 (27.3)
Race and ethnicity			
Hispanic	183 (39.4)	163 (41.0)	20 (30.3)
Black	64 (13.8)	51 (12.8)	13 (19.7)
White	185 (39.9)	159 (39.9)	26 (39.4)
Other^d^	32 (6.9)	25 (6.3)	7 (10.6)
Unstable housing status^e^	262 (57.8)	220 (56.6)	42 (65.6)
Medicaid health insurance	396 (85.3)	338 (84.9)	58 (87.9)
Navigator consultation in ED	387 (83.4)	345 (86.7)	42 (63.6)
Prior buprenorphine exposure	346 (74.6)	302 (75.9)	44 (66.7)
Any mental health condition^f^	328 (71.5)	279 (70.8)	49 (75.4)
Current substance use			
Fentanyl	242 (52.2)	200 (50.3)	42 (63.6)
Other opioids	391 (84.3)	333 (83.7)	58 (87.9)
Methamphetamine	232 (50.0)	189 (47.5)	43 (65.2)
Alcohol	140 (30.2)	109 (27.4)	31 (47.0)
Benzodiazepines or other sedatives	77 (16.6)	62 (15.6)	15 (22.7)
Cocaine or crystal cocaine	71 (15.3)	53 (13.3)	18 (27.3)

Abbreviation: ED, emergency department.

^a^ Unless indicated otherwise, values are presented as No. (%) of participants.

^b^ Sum may not equal total due to missing data.

^c^ Defined as buprenorphine administered or prescribed in the ED.

^d^ Includes American Indian or Alaska Native, Asian, Native Hawaiian or Other Pacific Islander, or multiple races or ethnicities.

^e^ Defined as not having one’s own house, apartment, or shared or rented housing.

^f^ Defined as at least 1 of the following: received inpatient psychiatric treatment in the past 30 days, any lifetime mental health diagnosis documented in the medical record, major depressive disorder likely (as measured with the 2-item Patient Health Questionnaire), or severe or moderate anxiety (as measured with the Generalized Anxiety Disorder 7-item scale).

**Figure 2.**
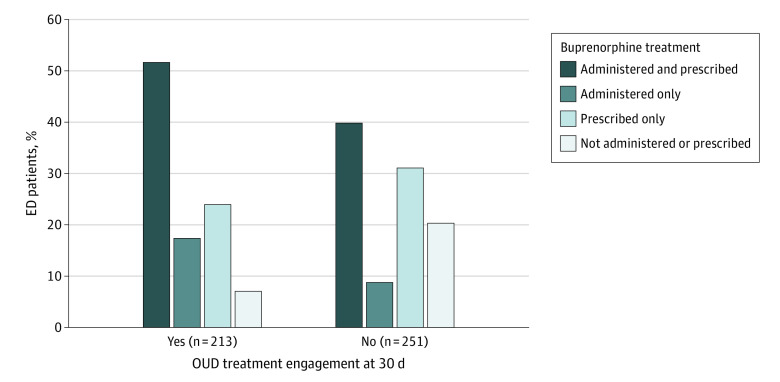
Opioid Use Disorder (OUD) Treatment Engagement at 30 Days by Emergency Department (ED) Buprenorphine Treatment Status OUD treatment engagement is shown for those who received buprenorphine in the ED vs those who did not (*P* < .001).

One month after the initial ED visit, 198 participants (49.7%) treated with buprenorphine in the ED were engaged in OUD treatment compared with 15 participants (22.7%) not treated with buprenorphine. The ED patients with OUD treated with buprenorphine were nearly twice as likely to be engaged in OUD treatment at 30 days (adjusted risk ratio, 1.97 [95% CI, 1.27-3.07]; Table 2). In analyzing how the 2 components of ED buprenorphine treatment varied by 30-day treatment engagement (Figure 2), the majority of participants (110 [51.6%]) engaged in OUD treatment were both administered and prescribed buprenorphine during the ED visit compared with 100 participants (39.8%) not engaged in OUD treatment. Of the remaining participants engaged in OUD treatment at 30 days, 37 (17.4%) were administered buprenorphine only, 51 (23.9%) were prescribed buprenorphine only, and 15 (7.0%) were neither administered nor prescribed buprenorphine during the ED visit compared with 22 (8.8%), 78 (31.1%), and 51 (20.3%) participants not engaged in OUD treatment at 30 days, respectively. Of the 213 participants engaged in treatment, engagement was confirmed with a clinically documented active prescription for buprenorphine for 196 (92.0%). Of the 339 patients prescribed buprenorphine in the ED, 206 (60.8%) had an active buprenorphine prescription at 30 days. A total of 17 of 213 participants (8.0%) were determined to be engaged based on self-report of participation in psychosocial treatment, methadone maintenance treatment, or both.

**Table 2.** Association of ED Buprenorphine Treatment With Subsequent OUD Treatment Engagement at 30 Days

ED buprenorphine treatment^a^	OUD treatment engagement at 30 d, No. (%) of patients		OR (95% CI)	
	Yes	No	Bivariate^b^	Adjusted^b^^,^^c^
Yes	198 (49.7)	200 (50.3)	2.19 (1.39-3.45)	1.97 (1.27-3.07)
No	15 (22.7)	51 (77.3)	1.00	1.00

Abbreviations: ED, emergency department; OR, odds ratio; OUD, opioid use disorder.

^a^ Defined as buprenorphine administered or prescribed in the ED.

^b^ Hierarchical generalized linear models include random effect for site as a group-level indicator.

^c^ Adjusted for age (years), sex, race and ethnicity, housing status, acceptance and availability of a patient navigator during the ED visit, and prior buprenorphine exposure.

Among the 66 participants who were not treated with buprenorphine, reasons included the following: declining buprenorphine because the participant was intending to engage in follow-up treatment but did not need acute treatment or a prescription, declining buprenorphine altogether as a treatment, and having a clinical contraindication such as the presence of severe illness, intoxication, or receipt of opioid agonist medications for pain. A total of 64 participants (13.8%) were lost to follow-up: 50 (12.6%) were treated with buprenorphine in the ED and 14 (21.2%) were not.

## Discussion

In this cohort study of ED patients with OUD, those treated with buprenorphine were almost 2 times as likely to be engaged in OUD treatment at 30 days after discharge compared with those not treated with buprenorphine. The high uptake of ED buprenorphine treatment and its association with OUD treatment engagement at 30 days highlight the success of the CA Bridge intervention and underscores the critical access point to OUD treatment that EDs can provide. These findings are notable given that our study population had a high prevalence of comorbid mental health conditions, self-reported fentanyl and methamphetamine use, and adverse social conditions including unstable housing and reliance on Medicaid insurance.

Our findings of 30-day OUD treatment engagement compare favorably with buprenorphine initiation in specialty office settings.^12,13^ Many of the traditionally expected steps—such as complex intake evaluations and treatment readiness assessment—in buprenorphine initiation are omitted in the CA Bridge low-threshold model that emphasizes “making the right thing the easy thing” through a flexible, harm-reduction approach that promotes convenience, comfort, and interpersonal engagement with patient navigators.^14^ Although the focus of this analysis was on the medication component of the CA Bridge intervention, patient navigation is an important bundled intervention. Patient navigators follow up with patients within 7 to 14 days of ED discharge, with a focus on identifying challenges to treatment engagement and working with patients to surmount these barriers. Site clinical champions and patient navigators are trained to facilitate coordinated care that links acute ED interventions with outpatient services to promote ongoing engagement in multidisciplinary treatment for OUD after the ED visit. The low OUD treatment engagement at 30 days among those not treated with buprenorphine emphasizes the need for additional interventions to reach this population. Individuals who chose not to receive ED buprenorphine treatment likely had more treatment challenges, as demonstrated by higher percentages of patients experiencing unstable housing, comorbid mental illness, and use of other substances, including alcohol, methamphetamine, cocaine, or benzodiazepines, compared with patients who chose to receive ED buprenorphine treatment. Additionally, the proportion of patients receiving ED buprenorphine treatment who had a consultation with a patient navigator was higher compared with those who did not receive ED buprenorphine treatment. Future studies are needed to describe the effect of patient navigation on both uptake of buprenorphine treatment among ED patients with OUD and subsequent engagement in treatment after discharge.

### Limitations

This study has some limitations. The high rate of acceptance of buprenorphine treatment suggests selection bias toward patients with interest in treatment and may bias the association between ED buprenorphine treatment and 30-day treatment engagement. Identification of patients with OUD was done through multiple strategies, and it was not practically possible to track all potential study participants with OUD in the high-volume ED setting with multiple clinical staff engaging patients along multiple clinical handoffs common in ED care. Some participants may have been clinically diagnosed with OUD or engaged in treatment that was not documented in their EHR or referred to the research team. Our findings are also subject to residual confounding due to potentially incomplete or incorrect control of confounders. Furthermore, there may be misclassification in the assessment of the primary outcome. Among the 17 of 213 participants (8.0%) without clinical documentation of an active buprenorphine prescription, self-report of engagement in treatment was not independently confirmed, and social desirability bias due to stigma and discrimination associated with substance use may have led some participants to falsely report engagement in treatment. Furthermore, achievement of the primary outcome does not mean that all patients had 30-day continuous OUD treatment, since not all patients who was engaged in treatment at 30 days had continually been taking buprenorphine for the full 30 days after the ED visit. Finally, the regional context of California could limit the generalizability of these findings to patients in other areas, as the study participants may not be representative of all ED patients with OUD.

## Conclusions

The findings of this cohort study suggest that among ED patients with OUD, treatment with buprenorphine may substantially improve the likelihood of sustained treatment engagement 1 month later. Future research should investigate techniques to optimize both the uptake and effectiveness of buprenorphine initiation in low-threshold settings such as the ED.

## Notes

Supplement 1. **eAppendix.** Data Sources

Supplement 2.
Data Sharing Statement
