# Supplementary material for: Emergency Department Access to Buprenorphine for Opioid Use Disorder
Source: JAMA Netw Open. 2024 Jan 29;7(1):e2353771. doi: 10.1001/jamanetworkopen.2023.53771 (PMC10825722; doi:10.1001/jamanetworkopen.2023.53771)
Supplement: Supplement 1. — eAppendix. Data Sources [file jamanetwopen-e2353771-s001.pdf]

Supplementary Online Content

Herring AA, Rosen AD, Samuels EA, et al. Emergency department access to buprenorphine for opioid use disorder. *JAMA Netw Open*. 2024;7(1):e2353771. doi:10.1001/jamanetworkopen.2023.53771

eAppendix. Data Sources

This supplementary material has been provided by the authors to give readers additional information about their work

© 2024 Herring AA et al. *JAMA Network Open.*

eAppendix. Data Sources

	Data Source	
	**Electronic Health Record**	**Self-report**
Age	x	
Gender		x
Race/ethnicity		x
Unstable housing	x	x
Health insurance	x	
Current substance use	x	x
Mental health history	x	x
Buprenorphine prescription history	x	x
Buprenorphine administered in the ED	x	
Buprenorphine prescription at ED discharge	x	
Navigator consult in ED	x	
Treatment referral during ED encounter	x	
OUD treatment engagement at 30 days	x	x

© 2024 Herring AA et al. *JAMA Network Open.*
