# Supplementary material for: Emergency Department Access to Buprenorphine for Opioid Use Disorder
Source: JAMA Netw Open. 2024 Jan 29;7(1):e2353771. doi: 10.1001/jamanetworkopen.2023.53771 (PMC10825722; doi:10.1001/jamanetworkopen.2023.53771)
Supplement: Supplement 2. — Data Sharing Statement [file jamanetwopen-e2353771-s002.pdf]

Data Sharing Statement

Herring. Emergency Department Access to Buprenorphine for Opioid Use Disorder. *JAMA Netw Open*. Published January 29, 2024. doi:10.1001/jamanetworkopen.2023.53771

Data
Data available: Yes
Data types: Deidentified participant data
How to access data: Allison D. Rosen at ADRosen@mednet.ucla.edu
When available: With publication

Supporting Documents
Document types: None

Additional Information
Who can access the data: researchers whose proposed use of the data has been approved
Types of analyses: Cohort analysis
Mechanisms of data availability: With investigator support, after approval of a proposal, and a signed data access agreement.
